# Delayed Metacomprehension Judgments Do Not Directly Improve Learning from Texts

**DOI:** 10.3390/jintelligence11070150

**Published:** 2023-07-24

**Authors:** Hannah Hausman, Veit Kubik

**Affiliations:** 1 Department of Psychology, University of California, Santa Cruz, CA 95064, USA; 2 Department of Psychology, University of Würzburg, 97070 Würzburg, Germany; veit.kubik@uni-wuerzburg.de

**Keywords:** JOL reactivity, judgments of learning, metacomprehension, covert retrieval

## Abstract

Making judgments of learning (JOLs) after studying can directly improve learning. This JOL reactivity has been shown for simple materials but has scarcely been investigated with educationally relevant materials such as expository texts. The few existing studies have not yet reported any consistent gains in text comprehension due to providing JOLs. In the present study, we hypothesized that increasing the chances of covert retrieval attempts when making JOLs after each of five to-be-studied text passages would produce comprehension benefits at 1 week compared to restudy. In a between-subjects design, we manipulated both whether participants (*N* = 210) were instructed to covertly retrieve the texts, and whether they made delayed target-absent JOLs. The results indicated that delayed, target-absent JOLs did not improve text comprehension after 1 week, regardless of whether prior instructions to engage in covert retrieval were provided. Based on the two-stage model of JOLs, we reasoned that participants’ retrieval attempts during metacomprehension judgments were either insufficient (i.e., due to a quick familiarity assessment) or were ineffective (e.g., due to low retrieval success).

## 1. Introduction

Metacognition refers to our thinking about our own thinking (Flavell 1979) and consists of two related processes: monitoring and control (e.g., Nelson and Narens 1990). Monitoring involves judging the state of one’s own learning, memory, understanding, knowledge, and skills. Control involves self-regulating one’s cognitive processes and learning behaviors as a result of monitoring. Metacognitive monitoring can indirectly improve learning via effective metacognitive control (Metcalfe 2009; Thiede and Dunlosky 1999; Winne and Hadwin 1998). For example, a student may accurately monitor their understanding, judging that they will not be able to answer questions about a particular concept on an upcoming exam. The student may then prioritize restudying that topic and understand it better as a result.

One of the most common measures of metacognitive processes are judgments of learning (JOLs), which typically ask people to judge the likelihood that they will be able to recall information on a future test (for a review, see (Rhodes 2016)). Not only can JOLs indirectly influence learning via metacognitive control, but JOLs can also directly affect learning of the information being judged, an effect termed *JOL reactivity* (Janes et al. 2018; Mitchum et al. 2016; Myers et al. 2020; Soderstrom et al. 2015; Witherby and Tauber 2017; for a meta-analysis, see (Double et al. 2018)). For example, Soderstrom et al. (2015) had participants study strongly related word pairs (e.g., *blunt–sharp*) and provide JOLs during the presentation of half the pairs, rating the likelihood they would be able to recall the pair. On the cued-recall test a few minutes later, participants recalled more targets when they had made JOLs for the pair during the study phase when they did not. Based on results like these, researchers have suggested that soliciting metacognitive judgments has the potential to directly improve learning in educational settings (Soderstrom et al. 2015; Witherby and Tauber 2017). 

However, there is currently no evidence that the direct learning benefits of JOLs extend to more educationally relevant materials as this has primarily been researched with simple materials, such as single word lists and word pairs (for a meta-analysis, see (Double et al. 2018)). Only three studies to our knowledge have examined how making metacognitive judgments directly affects learning with more complex, educationally relevant materials, and they did not find any evidence of JOL reactivity (Ariel et al. 2021; Schäfer and Undorf 2023; Trillo et al. 2022). Similar to Soderstrom et al.’ (2015) research with word pairs, Schäfer and Undorf (2023) presented general knowledge facts (e.g., *John Adams was the second U.S. president*) and half of the participants made JOLs while the fact was present on the screen, judging the likelihood they would remember it on the test. Participants recalled the facts at similar rates on the final test regardless of whether they made JOLs during the study.

Ariel et al. (2021) similarly did not find any evidence that making metacognitive judgments directly improves learning from texts. Across three experiments, participants read five brief sections of a text about geology and half of the participants made multiple term-specific judgements after reading each section (e.g., “How well do you understand what a crystalline solid is?”). Thus, unlike prior research on JOL reactivity, the participants judged their comprehension rather than predicting their literal recall of the presented information. We also refer to these metacomprehension judgments as JOLs for simplicity and the General Discussion addresses possible differences between typical judgments of learning and judgments of comprehension. Performance on the final test (e.g., Q: *What is a crystalline solid?* A: *A solid in which atoms are arranged in a regular, repeating pattern*) did not depend on whether participants had previously made JOLs (see also Trillo et al. 2022). In short, no existing research with complex educationally relevant materials has found any evidence that providing metacognitive judgments directly improves learning.

However, the way JOLs were solicited in prior studies may have limited the degree to which metacognitive judgments could directly improve learning of more complex materials. Participants provided JOLs with the questions and answers visible (Schäfer and Undorf 2023); they made judgments while initially studying the material (Schäfer and Undorf 2023) or immediately afterwards (Ariel et al. 2021; Trillo et al. 2022); and learning was measured on a final test that occurred immediately after the study phase (Ariel et al. 2021; Schäfer and Undorf 2023). Informed by theory and previous empirical findings, the present experiment examined JOL reactivity under more optimal conditions that should increase the likelihood that providing metacomprehension judgments would improve text comprehension. In the present study, JOLs were made in the absence of the correct answer, there was a delay between learning the materials and providing JOLs, and the final test occurred after a retention interval of 1 week. Each of these features should increase JOL reactivity. Therefore, the present research provided a stronger test of the educational utility of metacognitive judgments for directly improving learning. Finding no evidence of reactivity for text comprehension would significantly undermine previous claims that metacognitive judgments alone are an effective way to increase learning in school. 

### 1.1. Moderators of Metacognitive Accuracy and Reactivity

#### 1.1.1. Metacognitive Judgment Format

JOLs can be categorized as target-present or target-absent judgments. Target-present JOLs involve presenting the complete cue–target word pair or the question and the answer and—while that information is visible—asking participants to rate the likelihood they will remember the target or answer on a later test or rate how well they understand the answer. Target-absent JOLs, in contrast, involve presenting only the cue or question. Unlike target-present JOLs, target-absent JOLs are thought to elicit covert retrieval practice, encouraging participants to search their memory for the answer as part of the process of judging what they know and will remember later (Jönsson et al. 2012; Kimball and Metcalfe 2003; Nelson and Dunlosky 1991). Target-absent and target-present JOLs are therefore analogous to covert retrieval practice and restudy opportunities, respectively (e.g., Jönsson et al. 2014). The magnitude of JOL reactivity should be larger for target-absent than target-present JOLs at least after long retention intervals, since covert retrieval practice has been shown to directly strengthen memory compared to restudying (Carpenter et al. 2006, 2008; Kubik et al. 2020; Smith et al. 2013; but see Sumeracki and Castillo 2022; Tauber et al. 2018) and retrieval practice is a highly effective learning strategy, including for text materials (for a meta-analysis, see (Rowland 2014)). Schäfer and Undorf (2023) may not have observed JOL reactivity for general knowledge trivia questions because participants made target-present JOLs, likely limiting opportunities to engage in retrieval.

Ariel et al. (2021; Experiment 3) directly tested this hypothesis: Participants read five sections of a text, and after each section, they either proceeded to the next section without making JOLs, made target-present JOLs (e.g., “How confident are you that you understand that minerals are made by geological processes?”), or made target-absent JOLs (e.g., “How confident are you that you understand how minerals are made?”). Text comprehension was similar among the three groups, suggesting that target-absent JOLs are not necessarily sufficient to produce JOL reactivity. However, participants made JOLs for each section of the text immediately after reading that section. In addition to how the JOLs are solicited, when they are solicited can influence the accuracy of JOLs and their effects on learning. 

#### 1.1.2. Metacognitive Judgment Timing

Metacognitive judgments made during initial study or immediately afterwards are generally less accurate predictors of learning and tend to be associated with less learning than metacognitive judgments that are provided at least a few minutes after studying the material (for a meta-analysis, see (Rhodes and Tauber 2011)). Research on the metacognitive accuracy of delayed versus immediate JOLs suggests that target-absent JOLs are far less accurate when provided immediately after learning because the target information is highly accessible in memory when the JOL is provided, which makes the associated covert retrieval easier and makes the material feel better learned than it actually is (Dunlosky and Nelson 1992). Easy retrieval practice is generally less beneficial for learning than more difficult retrieval practice (Pyc and Rawson 2009), and the benefits of review tasks are generally smaller when the information being studied is highly accessible in memory at the time of study (Bjork and Bjork 1992). Thus, if providing JOLs elicits covert retrieval, JOL reactivity should be greater for delayed JOLs than immediate JOLs as longer lags between initial study and retrieval practice increase learning (Carpenter et al. 2009; Pyc and Rawson 2009, 2012; Rawson et al. 2015), including when retrieval practice is covert rather than overt (Kornell 2009). 

One reason that Ariel et al. (2021) did not observe a difference in reactivity for target-present and target-absent JOLs could be that the JOLs were provided immediately after reading each text rather than after a delay (e.g., after having read all the texts) as they were in the present study. To our knowledge, the present study is the first to examine JOL reactivity with educationally relevant material using delayed metacomprehension judgments, even though delayed metacomprehension judgments have long been known to improve metacomprehension accuracy with texts (e.g., Thiede et al. 2010; for a metaanalysis, see (Yang et al. 2023)) and other educationally relevant materials and in authentic educational settings (for a review, see (Hausman et al. 2021)).

#### 1.1.3. Retention Interval

The hypothesized role of covert retrieval processes in producing JOL reactivity implies that the timing of the criterial test should be a third moderating factor. The benefits of retrieval practice compared to no review or restudying have been shown to be larger after a delay of at least one day; on an immediate final test, restudying can yield better performance than retrieval practice (for a meta-analysis, see (Rowland 2014)). Previous research on JOL reactivity has predominantly used immediate final tests (but see Jönsson et al. 2012; Sundqvist et al. 2012; Trillo et al. 2022). In one of the only studies examining whether a retention interval moderates JOL reactivity, Sundqvist et al. (2012) had participants study Swahili–English translations four times or study all of the translations and then make cue-only JOLs for all of the translations before repeating the study task and JOL task again. Performance was higher in the study group than delayed JOL group for participants who completed the final cued-recall test after a five-minute retention interval. However, the reverse was true for participants who completed the final test after a 1-week retention interval. It is possible that JOLs did not directly enhance long-term retention of the translations, but rather, indirectly improved learning by increasing the effectiveness of the subsequent study opportunity of new materials (Kubik et al. 2022). Nevertheless, the results suggest that the memorial benefits of JOLs depend on the timing of the criterial test just as the benefits of retrieval practice do. 

Therefore, prior research using more complex, educationally relevant materials may not have observed any JOL reactivity, in part because the criterial test occurred within minutes of the initial learning phase (Ariel et al. 2021; Schäfer and Undorf 2023). The test in the present study occurred after a 1-week retention interval. If producing target-absent JOLs elicits covert retrieval, we would expect the benefits for text comprehension to emerge on this delayed test.

On the contrary, in a follow-up study very similar to Ariel et al. (2021), Trillo et al. (2022) had participants read five sections of a text and half of participants provided JOLs after each section. There was no evidence of JOL reactivity on a test either two days or 1 week after participants read the texts. However, in those studies, participants made target-absent JOLs immediately after each section. The present study tested the hypothesis that the conditions that increase the benefits of retrieval practice should also increase JOL reactivity. We expected that compared to only reading the texts, providing target-absent JOLs would improve performance on a test after a 1-week retention interval, specifically when JOLs were provided several minutes rather than immediately after reading each section. The 1-week retention interval also increases the ecological validity of our study, since students generally cannot study the material just minutes before an exam.

### 1.2. Covert Retrieval Prompts

Our prediction that delayed JOLs would enhance text comprehension measured on a test after a 1-week retention interval was based on the hypothesis that target-absent JOLs elicit covert retrieval (Jönsson et al. 2012; Nelson and Dunlosky 1991; Spellman and Bjork 1992). However, research suggests that JOLs do not spontaneously elicit covert retrieval practice, or at least not an exhaustive memory search. The two-stage model of JOLs (Son and Metcalfe 2005) suggests that when prompted to provide a JOL, people quickly assess cue familiarity and only attempt to covertly retrieve the target information for some items. If familiarity is too low, people quickly give low JOLs, bypassing a retrieval attempt because the target feels unrecallable (Metcalfe and Finn 2008; Son and Metcalfe 2005; Tauber et al. 2015). Furthermore, when familiarity is high, people may forgo a covert retrieval attempt because the high familiarity gives rise to the sense that the material is well known and is likely to be recalled later (Ariel et al. 2021; Tauber et al. 2018). Therefore, when JOLs are made primarily on the basis of familiarity, covert retrieval will be bypassed or truncated, and memory will not benefit as a result. Indeed, Son and Metcalfe (2005) found that encouraging participants to covertly retrieve prior to making JOLs improved final test performance compared to JOLs alone.

In short, target-absent JOLs likely invoke retrieval processes similar to retrieval practice, but JOLs likely involve a less thorough or effortful memory search (Rhodes and Tauber 2011; Tauber et al. 2015). We hypothesized then, that in the absence of direct instructions to thoroughly covertly retrieve the material prior to monitoring learning, JOLs would be based on high cue familiarity (Metcalfe and Finn 2008), general domain familiarity (Maki and Serra 1992), and/or ease of processing while reading the texts (Rawson and Dunlosky 2002). With direct instructions to engage in covert retrieval, we anticipated that participants would engage in exhaustive, more effortful retrieval attempts, thereby enhancing text comprehension. To test this hypothesis, half of participants were instructed to retrieve the texts they just read prior to making JOLs. If prompting participants to thoroughly, covertly retrieve the text before making delayed, target-absent JOLs does not enhance comprehension measured on a test after a 1-week retention interval, then the potential for JOL reactivity with educationally relevant materials would be seriously called into question.

Although our primary interest was whether JOLs directly enhance text comprehension, we also report relative metacomprehension accuracy, also known as *resolution* (Nelson 1984). Examining resolution has important theoretical and practical implications because the resolution of metacomprehension judgments tends to be quite low, meaning that learners struggle to differentiate between the topics or texts they have understood well and those they have understood more poorly (for a review, see (Griffin et al. 2019); for a meta-analysis, see (Yang et al. 2023)). If covert retrieval instructions prior to making JOLs encourages a more effortful and thorough memory search than JOLs alone, then covert retrieval instructions should increase relative metacognitive monitoring accuracy since retrieval success and retrieval fluency are typically more accurate indicators of memory strength and text comprehension than familiarity or ease of processing (Dunlosky and Nelson 1992). Therefore, we predicted that resolution would be higher among participants who received covert retrieval instructions than participants who did not (c.f. Thiede et al. 2003).

### 1.3. The Current Study

The present study investigated whether providing metacomprehension judgments directly enhances comprehension with educationally relevant expository texts. The goal was to test whether positive JOL reactivity emerges with a strong manipulation of learning conditions that should—based on theory and prior research—optimize the learning benefits of JOLs. Participants read five sections of a text (from Ariel et al. 2021; Trillo et al. 2022) and after they read all five sections, we factorially manipulated whether participants were instructed to covertly retrieve the text and whether they made delayed, target-absent JOLs. Participants in the retrieval + JOL group were first instructed to engage in covert retrieval practice and then they provided JOLs. Text comprehension was measured on a final test after a one-week retention interval.

We hypothesized that providing delayed JOLs involves the same fundamental retrieval processes as retrieval practice, but that retrieval practice involves a more comprehensive and effortful memory search than making JOLs. Thus, we hypothesized that engaging in one or both of the tasks would improve learning. We predicted final test performance would be lower in the no retrieval + no JOL group than the other three groups combined, and that the final test performance would be lower in the no retrieval + no JOL group than the retrieval + JOL group. Furthermore, we hypothesized each of the tasks would be beneficial on their own. We predicted higher final test performance in the no retrieval + JOL group than the no retrieval + no JOL group and higher final test performance in the retrieval + no JOL group than the no retrieval + no JOL group. Finally, based on research suggesting that covert retrieval instructions prior to making JOLs can encourage a more thorough memory search (Son and Metcalfe 2005), we predicted better test performance and higher relative metacognitive monitoring accuracy in the retrieval + JOL group than the no retrieval + JOL group.

## 2. Methods

The preregistration, materials, data, and R code are openly available at https://osf.io/evr5m (accessed on 15 May 2023).

### 2.1. Participants

Two hundred forty-eight participants were recruited from Prolific to complete the two-part study, which took approximately 25 min total, for USD 4.50. In total, 241 of the 248 participants completed both parts of the study. Participants were fluent English speakers and an average of 34 years old (*SD* = 11.27; range: 19–75; 25th percentile: 25; median: 32; 75th percentile: 40), and 65% were male, with one participant preferring not to report their sex. Most participants were living in the United Kingdom (43%), Poland (12%), Portugal (10%), and the United States (10%). 

#### 2.1.1. Exclusion Criteria

The participants were instructed not to take notes on the texts during the study phase and not to use notes or the internet to answer the test to answer test questions. Among the 241 participants who completed the experiment, 31 were excluded because they reported on the surveys at the end of the study phase and final test that they took notes or recorded the texts in some way, they used notes or the internet to answer to the test questions, or they were quite distracted during the study and did not give it much effort at all. Participants were told to answer these survey questions honestly and that their compensation would not be affected by their answers. Therefore, the final sample size after applying our preregistered exclusion criteria was 210. Participants were randomly assigned to one of four experimental groups based on a 2 (covert retrieval instruction: yes vs. no) × 2 (delayed JOLs: yes vs. no) factorial design: no retrieval + no JOL (*n* = 55), no retrieval + JOL (*n* = 49), retrieval + no JOL (*n* = 54), and retrieval + JOL (*n* = 52).

#### 2.1.2. Statistical Power

There is no prior research investigating the combined effects of delayed JOLs and covert retrieval activities on relative metacognitive monitoring accuracy and long-term retention of text materials. The preregistered initial sample size of 248 participants was therefore chosen to be consistent with the sample sizes (*n* = 60–65 participants per experimental group) in similar studies examining JOL reactivity with text materials (Ariel et al. 2021; Trillo et al. 2022). Our focal preregistered analyses were one-sided, independent samples *t*-tests. A sensitivity analysis of the final sample size revealed that *n* = 210, or approximately 53 participants per experimental group, is sufficient to detect group differences with *d* = 0.45 as the effect size with 80% power and α = .05 (G*Power, Version 3.1.9.2; (Faul et al. 2007)).

### 2.2. Materials

The materials were those used by Ariel et al. (2021). Participants read a text on minerals, divided into five sections with approximately 100 words each, with a Flesch reading ease score of 49.1 and a Flesch–Kincaid grade level of 10. Each of the sections had a descriptive heading (Geological Processes, Inorganic Substances, Crystalline Solids, Elements, and Compounds). The 12 short-answer test questions involved recalling information directly stated in the text (e.g., the test question “What are organic substances?” could be answered by the sentence in the text stating that “Organic substances are all the carbon-based compounds made by living creatures, including proteins, carbohydrates, and oils.”). There were 2–3 questions per section of the text. 

### 2.3. Procedure

**Figure 1:**
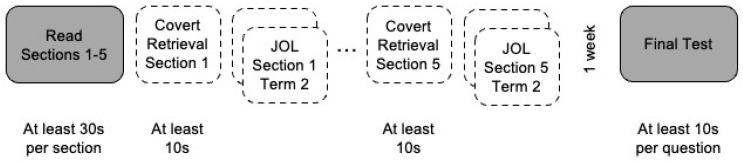
Experimental Procedure. ***Note.*** The gray boxes are tasks that every participant completed. The white boxes with dashed borders indicate tasks that participants completed depending on experimental group. Participants in the retrieval + JOL group and the retrieval + no JOL group completed the boxes labeled Covert Retrieval. Participants in the retrieval + JOL group and the no retrieval + JOL group completed the boxes labeled JOL, with each box referring to a JOL solicited for a different term from the corresponding section of the text. All tasks were self-paced, but some tasks had a minimum time requirement.

The experiment consisted of a study phase and a test phase two days later (Figure 1). Participants were randomly assigned to one of four experimental groups based on a 2 (covert retrieval instruction: yes vs. no) × 2 (delayed JOLs: yes vs. no) factorial design. During the study phase, participants read each of the five titled sections, one at a time, in a fixed order. Participants had to spend at least 30 s reading each section. The study phase ended for participants in the no retrieval + no JOL group or continued with covert retrieval practice, JOLs, or both, for participants in the remaining groups. 

Participants in the no retrieval + JOL group then provided 12 self-paced term-specific JOLs, 2–3 per text, on a sliding scale from 0 (*not very confident*) to 100 (*very confident*). The terms for which participants made JOLs corresponded to the terms on the test (e.g., “Rate how confident you are that you understood how minerals are made” corresponds to the test question “How are minerals made?”) and were presented in a fixed order for each participant based on the order they appeared in the text. After reading all five sections, participants in the retrieval + no JOL group were shown the title of each section of the text (e.g., *Geological Processes*), one at a time, in the order initially read. Participants were instructed to think back to the section and take as much time as they needed to recall as much of it as they could in their head, but they were required to spend at least 10 s covertly retrieving the section. Finally, participants in the retrieval + JOL group were first instructed to covertly retrieve a section, then they provided the 2–3 term-specific JOLs corresponding to that section on the next page and repeated this procedure for the remaining sections. The final test occurred 1 week after the study phase. The 12 short-answer test questions were presented in a fixed order, one at a time, corresponding to the order the information was presented in the text. Participants were required to spend at least 10 s answering each question.

### 2.4. Scoring and Analysis Plan

The final test short answer responses were scored as correct or incorrect by an experimenter blind to the participants’ group assignments. An answer was scored as correct if it matched the answer stated in the text or had the same meeting. For example, when the answer from the text was “atoms arranged in a regular repeating pattern,” the answer “atoms or molecules take up fixed repetitive patterns” was accepted. Thus, the factual final test questions assessed comprehension at the text-base level (Kintsch 1988). A second rater independently scored 17 randomly selected responses from each question. There was sufficient interrater reliability with *k* = .83, 95% CI [.77, .92], and discrepancies were resolved through discussion between the two raters. 

We preregistered directional predictions about the difference in means between two groups. Therefore, we conducted one-sided *t*-tests, implementing both classical null-hypothesis significance testing (NHST) procedures and Bayesian analyses. All analyses were conducted in R (v4.2.2; R Core Team 2022), relying on packages including Hmisc (v5.0-1; Harrell 2023), effectsize (v0.8.3; Ben-Shachar et al. 2020), rstatix (v0.7.2; Kassambara 2023), emmeans (v1.8.5; Lenth 2023), BayesFactor (v0.9.12-4.4; Morey and Rouder 2022), and bayestestR (v0.13.1; Makowski et al. 2019).

Bayesian analyses yield a Bayes factor, which quantifies the relative strength of the evidence for each hypothesis. For example, a Bayes factor of 10 indicates that the data are 10 times more likely under the alternative than the null hypothesis; a Bayes factor of 0.25 indicates that the data are 1/.25 or 4 times more likely under the null than the alternative hypothesis. The Bayes factor is denoted as *BF*_10_ for two-sided tests or *BF*_+0_ or *BF*_−0_ for right-tailed and left-tailed *t*-tests, respectively. When the data are more likely under the null than the alternative, we report the reciprocal for ease of interpretation, denoted *BF*_01_, or B*F*_0+_ or *BF*_0−_. Bayes factors are typically described as weak or inconclusive (1 < *BF* ≤ 3), moderate (3 < *BF* ≤ 10), strong (10 < *BF* ≤ 30, very strong (30 < *BF* ≤ 100), and extreme (*BF* > 100; Wagenmakers et al. 2018). Bayesian analyses also yield a posterior distribution, which conveys how probable different population effect sizes are after updating the prior based on observed data. The posterior distribution is often summarized by its median and the 95% highest density interval (*HDI*), which is the narrowest interval that captures the 95% most probable effect sizes. Our Bayesian analyses used the JZS prior centered at 0 with the default scale factor *r* = .707 (Wagenmakers et al. 2018), and we verified that the results were robust to the use of more diffuse priors.

## 3. Results

The participants spent approximately one minute reading each text and 20 s engaging in covert retrieval of each text. The magnitude of delayed JOLs was similar regardless of whether the participants were instructed to covertly retrieve first (Table 1). 

### 3.1. Metacognitive Resolution

The relative accuracy of JOLs was calculated separately for each participant as the Kruskal–Goodman gamma correlation between the JOL provided for each term and whether the final test question corresponding to that term was answered correctly. Gamma correlations range between −1 and +1, with more positive values indicating a better correspondence between metacognition and test performance (Rhodes 2016). Gamma could not be calculated for 11 participants who had invariance in their JOLs and/or test scores.

We predicted that covert retrieval prior to making JOLs would increase resolution. Gamma was numerically larger in the retrieval + JOL group (*M* = .21, *SD =* .43) than the no retrieval + JOL group (*M* = .16, *SD =* .47). However, a one-tailed independent samples *t*-test was not statistically significant and the effect size was small, *t*(88) = 0.47, *p* = .32, *d* = 0.10. The Bayes factor provided moderate evidence for the null hypothesis that the difference is zero relative to the alternative hypothesis that the difference is positive, *BF*_0+_ = 3.07. The posterior distribution^1^ of effect sizes was centered near zero (median = 0.09) and revealed a wide range of plausible effect sizes, from small negative to moderate positive effects, 95% *HDI* [−0.31, 0.47]. Thus, covertly retrieving prior to making JOLs did not improve relative metacognitive monitoring accuracy.

### 3.2. Text Comprehension

**Figure 2:**
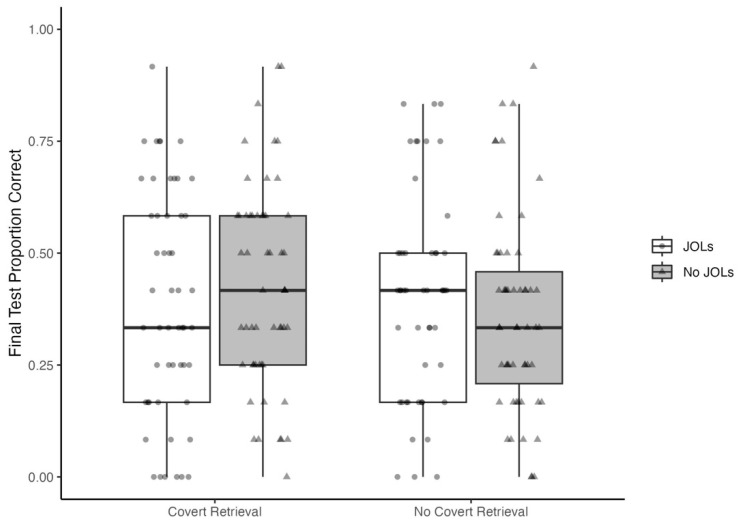
Proportion correct on the final test by experimental group.

Our remaining analyses pertained to the final test performance (Figure 2). Given the hypothesized benefits of covert retrieval practice and making metacognitive judgments, we predicted that receiving the instruction to engage in covert retrieval and/or providing delayed JOLs would produce better text comprehension than reading alone. To test this hypothesis, we conducted a 2 (covert retrieval instruction: yes vs. no) × 2 (delayed JOL: yes vs. no) factorial ANOVA of proportion correct on the final test with the contrast weight –1 for the no covert + no JOL group and 1/3 for each of the other three groups.^2^ Although raw proportion correct was numerically smaller in the no covert + no JOL group (*M* = .36, *SD* = .22) than the other three groups combined (*M* = .41, *SD* = .23), a one-sided test of this contrast revealed this difference was not statistically significant, *t*(206) = 1.25, *p* = .11, *d* = 0.20. Similarly, a one-sided Bayesian *t*-test supported the conclusion that there was no difference in means compared to the alternative hypothesis of the positive difference, *BF*_0+_ = 1.59, although the evidence was weak. The posterior distribution was centered near a small positive effect size (median = 0.18), but plausible effect sizes ranged from small negative to moderate positive effects, 95% *HDI* [−0.12, 0.48]. Thus, contrary to our prediction, engaging in covert retrieval and/or providing JOLs did not meaningfully enhance text comprehension.

#### 3.2.1. JOL Reactivity

We predicted that providing delayed metacognitive judgments would improve memory for the text on the test after a 1-week retention interval. Contrary to this prediction, a one-tailed *t*-test revealed no significant difference in the final test performance between participants in the no covert + JOL condition and the no covert + no JOL condition, *t*(102) = 0.96, *p* = .17, *d* = 0.19. The Bayesian analyses also suggested that JOLs did not enhance learning compared to reading alone, although the evidence was only weakly in favor of this null hypothesis, *BF*_0+_ = 1.95. The posterior distribution was centered near a very small positive effect size (median = 0.17), and small negative to moderate positive effects sizes were plausible, 95% *HCI* = [−0.19, 0.53]. Thus, we found no evidence for JOL reactivity in the absence of covert retrieval instructions.

#### 3.2.2. Covert Retrieval Practice Effects

Although not our primary focus, the experimental design also allowed us to test the prediction that delayed covert retrieval practice would enhance learning. Contrary to this prediction, a one-sided *t*-test revealed no significant difference in the final test performance between participants in the covert + no JOL condition and the no covert + no JOL condition, *t*(107) = 1.57, *p* = .06, *d* = 0.30. The Bayes factor suggests the data were equally likely under the alternative hypothesis of a positive effect compared to the null hypothesis of a no difference, *BF*_+0_ = 1.13. The posterior distribution was centered near a small positive effect size (median = 0.27), with the most probable effect sizes being near 0 or positive and moderate in size [95% *HCI* = [−0.07, 0.64]. Thus, we found no evidence for covert retrieval practice effects.

#### 3.2.3. Effects of Covert Retrieval on JOL Reactivity

Given the hypothesized benefits of prior covert retrieval instructions and making metacognitive judgments, we predicted that engaging in covert retrieval and providing delayed JOLs would produce better text comprehension than reading alone. Contrary to this prediction, a one-sided *t*-test revealed no significant difference in final test performance between participants in the retrieval + JOL condition and the no retrieval + no JOL condition, *t*(105) = 0.60, *p* = .28, *d* = 0.12. The Bayesian analyses also suggested that covert retrieval prior to making JOLs does not enhance learning beyond reading alone, *BF*_0+_ = 3.89, posterior median = 0.10, 95% *HCI* = [−0.25, 0.46]. Thus, we found no evidence for JOL reactivity even when participants who made JOLs were encouraged to thoroughly retrieve first.

We also predicted prior to collecting data that encouraging more thorough retrieval prior to making JOLs would amplify JOL reactivity. Although the previous analyses suggest otherwise, we report the analysis for completeness. A one-sided *t*-test revealed no significant difference in the final test performance between participants in the retrieval + JOL group and the no retrieval + JOL group, *t*(99) = –0.32, *p* = .06, *d* = –0.06, and the test performance was numerically greater in the no retrieval + JOL group (Table 1). The Bayesian analyses also suggest that a covert retrieval instruction prior to making JOLs did not enhance learning from making JOLs, *BF*_0+_ = 8.32, posterior median = –0.06, 95% *HCI* = [−0.43, 0.30]. 

### 3.3. Exploratory Analyses

We predicted that providing JOLs would enhance memory for the text by eliciting covert retrieval practice. Longer times spent making JOLs may indicate that participants spent more time engaging in covert retrieval prior to providing their judgments and may therefore be associated with better text comprehension. We calculated the Pearson correlation between total time providing JOLs and final test performance among participants in the no retrieval + JOL group only; JOL times would be difficult to interpret in the retrieval + JOL group because JOLs were provided after covert retrieval instructions. JOL times and final test performance were positively associated, *r* = 0.39, and a two-tailed one-sample *t*-test revealed that this correlation was significantly different from zero, *t*(47) = 2.88, *p* = .006, *d* = 0.84. The Bayesian analysis of the Pearson correlation using a beta distribution prior centered with a scale factor of 1/3 revealed moderate-to-strong evidence that time spent providing JOLs was associated with test performance, *BF*_10_ = 9.52, posterior median = 0.35, 95% *HDI* [0.10, 0.57]. 

However, covert retrieval time was not associated with better text comprehension. We calculated the Pearson correlation between total time providing JOLs and proportion correct on the final test among participants in the retrieval + no JOL group, which was small and not statistically significant, *r* = 0.15, *t*(52) = 1.10, *p* = 0.23, *d* = 0.31. The Bayesian analysis of this correlation revealed weak evidence in support of the null hypothesis of no correlation, *BF*_01_ = 1.90, and the posterior distribution of the true population correlation was centered near 0 (median = 0.13), with both negative and positive correlations being plausible after taking the observed data into account, 95% *HCI* [−0.13, 0.37].

## 4. Discussion

Prior research with simple materials such as single word lists and word pairs has found that explicitly providing metacognitive judgments can directly affect the learning of the information being judged and, when it does, typically improves learning (Double et al. 2018). Despite these initial signs that metacognitive judgments could be a tool to directly benefit learning in school, the results of prior JOL reactivity research with more educationally relevant materials were not promising (Ariel et al. 2021; Schäfer and Undorf 2023; Trillo et al. 2022).

### 4.1. JOL Reactivity Terminology

To facilitate the interpretation of the present study in the context of past research, and to support the development of future research, we suggest the following standardized terminology. We propose that JOLs should be the overarching term to refer to metacognitive judgments in which individuals are asked to self-assess their learning and that researchers should specify different types of JOLs as needed. JOLs have typically asked participants to rate the likelihood they will recall a specific piece of information or answer a specific type of question correctly on an upcoming test (e.g., Rhodes 2016). These could be referred to as JOLs broadly, or predictions of recall (PORs), specifically. In the present study, the metacognitive judgments prompted participants to rate their understanding of ideas from the text (e.g., “How well do you understand what a crystalline solid is?”), rather than predict their ability to recall the fact or definition on in the future (e.g., “How likely are you to recall what a crystalline solid is on the test?”). Thus, we suggest that participants also made JOLs in the present study, and specifically, their JOLs were judgments of comprehension (JOCs). JOCs were the type of JOL used in prior reactivity research with texts that this study extends (Ariel et al. 2021; Trillo et al. 2022). We propose the term JOL reactivity be used to refer to the finding that providing metacognitive judgments directly affects learning, regardless of the type of materials (e.g., related word pairs, trivia, or texts), type of JOL (e.g., POR or JOC; target-present or target-absent; and immediate or delayed), and regardless of how learning is assessed on a criterial test (e.g., cued recall or free recall; factual questions or inference questions).

### 4.2. The Present Study

We hypothesized that prior studies found no JOL reactivity with general knowledge facts (Schäfer and Undorf 2023) or texts (Ariel et al. 2021; Trillo et al. 2022) because JOLs were provided with the target present, while participants were initially learning the material or immediately afterwards, and/or the final test occurred immediately after learning. We predicted that conditions that make covert retrieval more likely while participants made JOLs would increase learning from the text, as suggested from prior work with simpler materials (Carpenter et al. 2006, 2008; Kubik et al. 2022; Smith et al. 2013; but see Tauber et al. 2018).

Contrary to our predictions, the present study similarly revealed that JOLs did not improve text comprehension. This outcome was surprising given that the present study used learning and test conditions that should have maximized the benefits of JOLs for learning, according to prior research and theory. JOLs were made with the target information absent (Jönsson et al. 2012; Kimball and Metcalfe 2003; Nelson and Dunlosky 1991), and following a brief delay, after the participants had read all the texts, rather than after each text (for a meta-analysis, see (Rhodes and Tauber 2011)). The final test also occurred after a 1-week retention interval given that learners reap the benefits of covert retrieval after long delays (for a meta-analysis, see (Rowland 2014)). Prior research with text materials has revealed that the benefits of retrieval practice over restudying are even larger after a 1-week than 2-day delay (e.g., Roediger and Karpicke 2006). Finally, the no JOL control group was only exposed to the material once and did not reread the texts, while participants in the other groups were given covert retrieval instructions or JOL prompts. Thus, the present study provided the strongest test to date of whether providing target-absent JOLs can directly enhance the learning of educationally relevant material, and we found that it does not. Compared to only reading the texts once, providing delayed, target-absent JOLs did not enhance comprehension on the delayed final test.

Based on the hypothesis that delayed, target-absent JOLs elicit covert retrieval practice, there are at least two explanations for why we did not find JOL reactivity in the present study, which we refer to as the *insufficient-retrieval* and the *ineffective-retrieval explanations*. These explanations are not mutually exclusive; insufficient retrieval may explain why JOLs did not directly enhance learning for some items or some participants, whereas ineffective retrieval may explain the lack of JOL reactivity for other items or participants. Both explanations are consistent with the finding that covert retrieval instructions do not always lead to as much learning as overt retrieval attempts (Jönsson et al. 2014; Kubik et al. 2022; Sumeracki and Castillo 2022; Tauber et al. 2018).

### 4.3. Insufficient Retrieval

The *insufficient-retrieval explanation* suggests that we found no JOL reactivity because participants did not automatically covertly search their memory for relevant information, or at least not exhaustively, when providing their judgments (Ariel et al. 2021; Dunlosky et al. 2005; Metcalfe and Finn 2008; Son and Metcalfe 2005). The two-stage model of JOLs posits that people make JOLs by first quickly assessing the familiarity of the material; at moderate levels of familiarity, people will then search memory for the answer to inform their judgments (Metcalfe and Finn 2008; Son and Metcalfe 2005). Our exploratory correlation analyses revealed that participants who spent longer providing JOLs answered a greater proportion of questions correctly on the final test, which is consistent with the two-stage model of JOLs as well as the insufficient retrieval explanation for why we found no JOL reactivity. That we found no evidence of JOL reactivity on average, though, suggests participants generally did not spend sufficient time searching memory prior to making their JOLs, and this may be due to the type of JOL used. Certain types of metacognitive judgments, even when solicited without the target present and after a delay, may be less likely than others to trigger participants to automatically, covertly retrieve what they previously learned.

#### 4.3.1. Type of Metacognitive Judgment

There is evidence that JOCs and PORs (typically referred to as JOLs in prior research) involve different cognitive processes. Maki and Serra (1992) had participants read brief expository texts and predict their upcoming test performance or rate their understanding of the texts. Predictions of performance were better correlated with actual test performance than JOCs because participants relied too heavily on domain familiarity rather than an actual understanding of the specific material in the text when making JOCs (see also Maki et al. 1990). 

Based on the two-stage model of JOLs, JOCs may be particularly likely to involve quick judgments based on familiarity that bypass retrieval. In the case of complex, causal processes like those explained in the texts in the present study, people tend to overestimate their depth and specificity of understanding (Alter et al. 2010; Rozenblit and Keil 2002). The phrasing of PORs in terms of one’s ability to recall information on a test might cause learners to consult their memory for what they read rather than a more gist-based sense of what they know. Even if JOCs elicit a retrieval attempt, participants may terminate their memory search early, mistakenly believing they have recalled all the relevant information. Indeed, when answering questions overtly, participants are prone to overestimating the comprehensiveness and accuracy of their answers (Dunlosky et al. 2005; Dunlosky and Rawson 2012; Lipko et al. 2009; Rawson and Dunlosky 2007; Zamary et al. 2016). Future research should examine whether and how the type of metacognitive judgment—POR vs. JOC—affect reactivity. 

#### 4.3.2. Limited Benefits of Covert Retrieval Instructions

One reason for the lack of JOL reactivity in the present study is that the delayed, target-absent JOLs did not elicit sufficiently thorough retrieval attempts. If this is the case, additional covert-retrieval instructions prior to making JOLs should have improved text comprehension on the final test 1 week later (cf. Son and Metcalfe 2005). However, we did not find any evidence for such a finding, which seems to be inconsistent with the insufficient-retrieval explanation. Like the JOL prompt, merely instructing participants to engage in covert retrieval may not have elicited an extensive memory search. For relatively complex, educationally relevant materials, participants may attempt covert retrieval but not recall all the relevant pieces of information (Sumeracki and Castillo 2022; Tauber et al. 2018). Supporting this explanation, prior research has found with key-term definitions or texts that overt but not covert retrieval prior to making target-absent JOLs increases learning compared to only making target-absent JOLs (Ariel et al. 2021; Tauber et al. 2018). This could also explain why comprehension and metacognitive resolution were not better in the covert + no JOL group than the no covert + no JOL group.

In sum, the insufficient-retrieval explanation proposes that participants had information from the text accessible in long-term memory, but they did not retrieve it when making JOLs or following the covert retrieval instructions because they did not engage in retrieval or terminated their memory search early due to overreliance on familiarity. Participants would not reap the benefits of retrieval practice for the known but unretrieved information (Son and Metcalfe 2005), resulting in limited JOL reactivity.

### 4.4. Ineffective Retrieval

The *ineffective-retrieval explanation* is that participants attempted relatively extensive memory searches when providing JOLs or following the covert retrieval instructions but that these memory searches did not improve text comprehension. Like overt retrieval, the mnemonic benefit of covert retrieval may depend on retrieval success (Rowland 2014). When feedback is not provided, information can only be strengthened in memory by a retrieval attempt if it is successfully retrieved. Rowland’s (2014) meta-analysis revealed that retrieval practice only enhanced learning compared to restudy when feedback was not provided if more than 50% of the material was successfully retrieved. Even if participants in the present study exhaustively searched memory for what they previously read in the text, they may not have been able to successfully retrieve a large enough proportion of the texts to meaningfully increase comprehension on the final test. This could explain why our exploratory analyses found no correlation between time spent on covert retrieval and final test performance in the covert + no JOL group. 

In short, the insufficient-retrieval explanation attributes the lack of target-absent JOL reactivity or covert retrieval benefits to a failure to retrieve information that is accessible in long-term memory; the ineffective-retrieval explanation attributes our null results to participants attempting to retrieve information that is not accessible in long-term memory. Future studies could use various self-report measures of retrieval success as imperfect estimates of the extent of the covert memory searches and the amount of information correctly covertly retrieved (Tauber et al. 2018).

Although the evidence is indirect, existing research on JOL reactivity is more compatible with the ineffective-retrieval than the insufficient-retrieval explanation. If JOL reactivity depends on engaging in a thorough memory search, then JOL reactivity should be largely eliminated with target-present JOLs. Contrary to this prediction, most research on JOL reactivity has used target-present JOLs. To our knowledge, the only exceptions are the present study, and the similar preceding work on JOL reactivity with text materials (Ariel et al. 2021; Trillo et al. 2022). Other previous research using simpler materials such as related word pairs and word lists has reliably found JOL reactivity using target-present JOLs (Double et al. 2018). One of the leading explanations of JOL reactivity (with target-present JOLs) is that providing JOLs enhances processing of the cues that inform metacognitive judgments and final test performance will be improved when those same cues also facilitate subsequent recall. For example, when providing a JOL for the pair *dull*–*knife*, people attend to pair relatedness (e.g., Koriat 1997). Soderstrom et al. (2015) proposed that attending to metacognitive cues such as relatedness will increase encoding of the cue–target association (e.g., imagining a dull butter knife slicing through a stick of butter) more so than studying the pair without making a JOL, thereby improving memory on a cued recall test (see also Myers et al. 2020).

This theory could easily be extended to JOLs for more complex text materials. Asking “How well do you understand why water is not a mineral?” may be unproductive for remembering the correct answer (*because it is a liquid*) on the final test if participants cannot retrieve relevant information. In contrast, asking participants “How well do you understand that water is not a mineral?” provides the opportunity for something akin to elaboration or self-explanation, both of which are at least moderately effective for learning from texts compared to passive reading and rereading (for a review, see (Dunlosky et al. 2013)). Indeed, Ariel et al. (2021) found that target-present JOLs improved text comprehension more than target-absent JOLs, although the effect was relatively small and needs to be replicated in comparison to no JOL conditions. Although retrieval is a particularly effective way to strengthen memory when the information successfully retrieved, target-present JOLs may be more effective for learning complex materials such as texts, on average, because they provide an opportunity to review and elaborate on the material that is unlikely to be fully, successfully covertly retrieved during target-absent JOLs. If future research reveals that target-present JOLs improve text comprehension compared to target-absent JOLs and no JOLs, it will be important to control for total study time to disentangle the effects of making JOLs and additional exposure to the material.

### 4.5. Two-Factor Account of JOL Reactivity

We propose a new two-factor account of JOL reactivity, which suggests that metacognitive judgments will directly enhance learning to the degree that JOLs facilitate (1) successful, complete covert retrieval, (2) the elaborative encoding of cue–target associations, or both. The present study was formulated based on the hypothesis that delayed, target-absent JOLs elicit covert retrieval and thus was focused on the first mechanism in our two-factor model. As noted above, the majority of JOL reactivity research has attributed the memorial benefits of immediate, target-present JOLs to the second mechanism, elaboration of the cue–target relationship.

However, the covert retrieval and elaboration mechanisms for JOL reactivity are not mutually exclusive and are not specific to target-absent and target-present JOLs, respectively. For example, when a target-absent JOL elicits the successful covert retrieval of the target information, participants may further elaborate on what they retrieved (e.g., Finn 2017). Similarly, a target-present JOL may directly improve learning not only via elaborative encoding, but also by eliciting the covert retrieval of the initial learning phase (e.g., Benjamin and Ross 2010). The degree to which the covert retrieval and elaboration mechanisms contributes to JOL reactivity likely depends on the learning materials, the type and timing of the JOLs, and the type of test. Future research should systematically delineate the factors that affect JOL reactivity and the degree to which these factors exert their influence via covert retrieval and elaboration mechanisms.

In the present study, JOLs did not enhance text comprehension, which was attributed to the failure of the first mechanism in our two-factor model of JOL reactivity, that is, covert retrieval. Delayed, target-absent JOLs may not have elicited covert retrieval, or at least not a thorough memory search (insufficient retrieval). Alternatively, participants may have attempted to retrieve relevant prior information but did not benefit from these retrieval attempts because they were unsuccessful (ineffective retrieval). In either case, because we used target-absent JOLs, there was no opportunity for the JOLs to enhance learning via the second mechanism, that is, elaborative encoding. Thus, we found no JOL reactivity. We note that the present study did not directly test the two-factor model, so it should be treated as a proposal and systematically researched going forward. 

### 4.6. Limitations

Although the present study used educationally relevant texts, the participants were not students learning the material for a course. They did not have an incentive to perform well on the final test, for either a higher course grade or better pay. Thus, it is unclear whether the results would generalize to educational settings. Future research should examine JOL reactivity in authentic courses with genuine reading assignments and material that students need to learn for an exam. Another important consideration is whether any effects of JOLs on learning emerge when students can review the material between providing JOLs and taking the final test, as they typically can in real life.

## 5. Conclusions

The present study provided the strongest test to date of JOL reactivity with complex text materials. To our surprise, providing delayed, target-absent JOLs did not enhance comprehension on a test after a 1-week retention interval. Future research will need to systematically investigate different theoretical explanations for why the JOL reactivity that has been observed with simpler materials such as related word pairs and word lists does not appear to generalize to texts (Ariel et al. 2021; Trillo et al. 2022) and general knowledge statements (Schäfer and Undorf 2023). Thus, in the meantime, there is accumulating evidence that instructors and students should use caution if relying on target-absent JOLs to directly improve the learning of more complex materials. This seems particularly important because we can imagine a myriad of situations in which students monitor their learning similarly to how JOLs were solicited in the present study. For example, students preparing for an exam may read over the study guide and ask themselves how well they understand the terms and questions. Although a student may perceive this activity as studying, the present results suggest that it will not improve performance on the exam, at least not directly (see also Ariel et al. 2021; Schäfer and Undorf 2023; Trillo et al. 2022).

This is not to say that JOLs and other types of metacognitive monitoring judgments are not valuable learning tools. There is compelling evidence that supporting students in making accurate metacognitive judgments indirectly improves text comprehension and the learning of other educationally relevant materials via effective self-regulated study choices (e.g., Little and McDaniel 2015; for a review, see (Hausman et al. 2021)). However, if an instructor’s goal is to directly improve the learning of a course content, we suggest spaced, overt retrieval practice opportunities with feedback (for a recent overview, see (Carpenter et al. 2023)).

## Figures and Tables

**Table 1:** Mean (*SD*) of study and test phase measures by experimental group.

		Reading Time per Passage (s)	Covert Retrieval Time per Passage (s)	JOL	Time per JOL (s)	Final Test Proportion Correct
no retrieval	no JOL	57.13 (37.61)				.36 (.22)
no retrieval	JOL	51.87 (18.75)		66.07 (20.96)	10.90 (5.95)	.40 (.23)
retrieval	no JOL	54.15 (23.55)	23.65 (13.61)			.43 (.22)
retrieval	JOL	58.02 (31.55)	20.15 (8.67)	66.13 (22.90)	10.63 (5.98)	.39 (.25)

## Data Availability

The preregistration, materials, data, and R code can be accessed at https://osf.io/evr5m (accessed on 15 May 2023).

## Notes

1 Even for the one-sided *t*-tests, we report the median and *HDI* of the posterior distribution for effect sizes using a two-sided prior. This is preferable because it conveys the most probable effect sizes, not just positive ones (Wagenmakers et al. 2015).

2 Note this is a slight deviation from our preregistered one-way ANOVA of final test scores with condition as a factor with four levels. The contrast analysis is equivalent, though, regardless of ANOVA specification. The contrast weights differ from the preregistration, too, which were incorrect for our predicted hypothesis.
